# Supplementary material for: Spectroscopic evidence of odd frequency superconducting order
Source: Sci Rep. 2017 Jan 20;7:40604. doi: 10.1038/srep40604 (PMC5247762; doi:10.1038/srep40604)
Supplement: Supplementary Information [file srep40604-s1.doc]

**Supplementary information**

**Spectroscopic evidence of odd frequency superconducting order**

Avradeep Pal^1*^­­, J. A. Ouassou^2^, M. Eschrig^3^, J. Linder^2*^, M. G. Blamire^1^

*^1^Department of Materials Science, University of Cambridge, 27 Charles Babbage Road, Cambridge CB3 0FS, United Kingdom ^2^Department of Physics, Norwegian University of Science and Technology, N-7491 Trondheim, Norway ^3^SEPnet and Hubbard Theory Consortium, Department of Physics, Royal Holloway, University of London, Egham, Surrey TW20 0EX, United Kingdom*

We provide the following evidence to rule out superconductivity in the TiN layer and establish conclusively that the ZBCP is due to SIN junctions and therefore represents an enhancement in DOS around the Fermi level in NbN:

1. **Variation of gap edge with temperature**

If TiN turned superconducting at 3K (
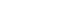
), and assuming that TiN behaves like an ideal BCS superconductor,
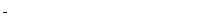
.

At 1.6K:
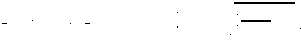
 = 0.42mV.

Supplementary Figure 1 – Differential conductance curves at temperatures from 4.5K to 1.6K demonstrating that there is no observation in enhancement of gap edge due to possible superconductivity of TiN layer.

Hence we would expect the gap edge in NbN/GdN/TiN junctions to increase by ~ 0.5 mV as the junction is cooled. The figure below and the inset to it, clearly indicates that no such increase is observed. In fact, no increase is observed even at 0.3K (Refer to Fig. 2 of the main manuscript). This is a clear demonstration that NbN is the only superconducting layer in the tri-layer stack. Owing to high transition temperature of NbN (
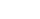
~13.5K), the magnitude of its gap edge feature saturates below approx. 7K. Thus, these junctions are SIN junctions throughout the entire temperature range below 13.5K.

1. **Discussion on a potential Josephson effect**

Josephson junctions with two superconducting NbN electrodes  [1] with exactly identical junction dimensions to the devices reported here exhibit *I_c_*(*H*) Frauhofer patterns with an oscillation period or lobe width of of 1.5-3.5 mT. If we assume that, despite the arguments above, that the TiN is superconducting then we can take its penetration depth (
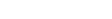
, and therefore should have lobe width:


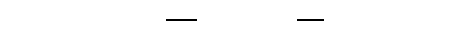


where
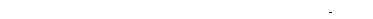
and
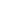
 is the universal flux quantum. As per the above expression – the Fraunhofer patterns should have critical current oscillation periods as approx. 4.4mT. Taking the standard decay envelope of the Fraunhofer pattern, we can therefore say that any Josephson effects should die out at high fields (>45mT; i.e. – beyond the supposed 10^th^ Fraunhofer lobe). However, the ZBCPs in our samples are only suppressed slowly and monotonically and remain visible even till 1 T, thus ruling out ZBCPs due to critical currents (conductance maxima) at low bias due to Josephson effects.

Supplementary Figure 2 – Differential conductance measurements of the ZBCP at different values of externally applied in-plane magnetic fields.

1. **Comparison of IV curves of SIS and SIN junctions**

Supplementary Figure 3 – IV curves (including forward and backward traces) of two junctions, one with a superconducting NbN counter-electrode (red), while the other has non-superconducting TiN electrode (blue). IV curves are measured at zero field and well below the supposed superconducting Tc of NbN (13.5K) and TiN (either 5.4K or 3K as suggested by referee). Inset to figure shows the comparison of respective differential conductance curves measured with an in-plane magnetic field of 10mT.

The most noticeable features in Figure 3 are that for the (SIN) junction with the TiN counter-electrode (blue curve), the high conductance region around zero bias is not vertical and that switching away from this region (which occurs at roughly the same magnitude of current (
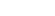
) as the critical current of the NbN/GdN/NbN SIS junction) shows no sign of hysteresis – a feature that is expected in underdamped SIS Josephson junctions and clearly present in the NbN/GdN/NbN device. However, for the junction with NbN counter-electrode, we see clear hysteresis (Switching current
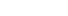
 Re-trapping current
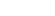
). We have reported in a prior publication  [2] that our GdN junctions are in the underdamped regime, and hence the above IV traces clearly demonstrate that the high conductance region in TiN junctions is not a feature caused by critical currents, but rather, represents an enhancement in the DOS around the Fermi level of the NbN superconductor for the NbN/GdN/TiN device.

The inset shows the previously discussed effect of magnetic field on the supercurrent due to the Josephson Effect (Fraunhofer pattern). While the critical current in NbN/GdN/NbN junction is completely suppressed due to the 10 mT in-plane magnetic field; an identical in-plane field has no effect on the magnitude of the ZBCP.
